# Therapeutic Potential of Luteolin on Impaired Wound Healing in Streptozotocin-Induced Rats

**DOI:** 10.3390/biomedicines9070761

**Published:** 2021-06-30

**Authors:** Li-You Chen, Hsin-Lin Cheng, Yu-Hsiang Kuan, Tang-Jun Liang, Yun-Yi Chao, Hsing-Chun Lin

**Affiliations:** 1 Department of Anatomy, School of Medicine, College of Medicine, Chung Shan Medical University, Taichung 40201, Taiwan; peiyu@csmu.edu.tw (L.-Y.C.); skym122951703@gmail.com (T.-J.L.); catherine2880343@gmail.com (Y.-Y.C.); 2 Department of Medical Education, Chung Shan Medical University Hospital, Taichung 40201, Taiwan; 3 Department of Nutrition, Chung Shan Medical University, Taichung 40201, Taiwan; iamsamlee@livemail.tw; 4 Department of Pharmacology, School of Medicine, Chung Shan Medical University, Taichung 40201, Taiwan; kuanyh@csmu.edu.tw; 5 Department of Pharmacy, Chung Shan Medical University Hospital, Taichung 40201, Taiwan; 6 Department of Nutrition, Chung Shan Medical University Hospital, Taichung 40201, Taiwan

**Keywords:** luteolin, diabetes, wound healing, anti-oxidation, anti-inflammation

## Abstract

Long-term hyperglycemia may lead to diabetic microvascular and macrovascular complications that can affect the peripheral vascular system, particularly in wound healing capacity. Impaired angiogenesis and delayed wound healing are significant clinically. Luteolin (3′, 4′, 5, 7-tetrahydroxyflavone) is a naturally occurring flavonoid that is ubiquitously found in plants. Recent evidence has shown that luteolin is an anti-inflammatory and anti-oxidative agent. However, the effect of systemic luteolin administration on diabetic wound restoration remains unclear. Herein, we explored the effectiveness of luteolin for improving delayed and impaired healing of skin wound and further clarified the underlying mechanisms. The results indicated that luteolin significantly attenuates blood glucose concentration, improves impaired healing and accelerates re-epithelization of skin wound in streptozotocin (STZ)-induced diabetic rats. Histopathological staining and immunoblotting revealed an inhibitory effect of luteolin on inflammatory cell and cytokine production. We also observed remarkable decreases in protein expressions of inflammatory factors including matrix metalloproteinase (MMP)-9, tumor necrosis factor (TNF)-α, interleukin (IL-6), and IL1-β and downregulation of nuclear factor (NF)-κB, as well as increases in anti-oxidative enzymes such as superoxide dismutase 1 (SOD1) and glutathione peroxidase (GSH-Px) induced by nuclear factor erythroid 2-related factor (Nrf)-2 following luteolin supplementation. Furthermore, luteolin decreased the expression of vascular endothelial growth factor (VEGF) and increased the expression of ubiquitin carboxy-terminal hydrolase (UCH)-L1, as evidenced by angiogenesis and neuronal regeneration in completely healed wound. In conclusion, systemic administration of luteolin promotes wound restoration by ameliorating inflammation and oxidative stress through the inactivation of NF-κB and upregulation of Nrf2 in STZ-induced diabetic rats.

## 1. Introduction

Diabetes mellitus (DM) is a group of chronic metabolic disorders that are characterized by hyperglycemia, defective insulin secretion and/or insulin action and strong correlation with higher risk of disease-related complications [1]. Long-term hyperglycemia in patients with diabetes confers a higher risk for chronic complications due to accumulations of toxic substances in tissues, which are irreversible and lead to microvascular (retinopathy, nephropathy, and neuropathy) or macrovascular (coronary heart disease, peripheral vascular disease, and stroke) diseases [2,3,4]. The significant physiological impairment associated with hyperglycemia has been traditionally linked to impaired wound healing. Prolonged hyperglycemia causes endothelial dysfunction, leading to downregulation of pro-angiogenic signature and induction of oxidative stress [5]. Wound healing is a multifaceted dynamic repair process of skin barrier breach that involves significant physiological events such as growth factors and cytokines in response to cellular immunity. In addition to immune response activation, impaired wound healing leads to overproduction of matrix metalloproteinases (MMPs), resulting in extracellular matrix degradation and failure to generate skin re-epithelialization [6,7,8]. Although the comprehensive knowledge of the pathological process of diabetes has been well clarified, commercially available therapeutics mainly focus on the transient hypoglycemic effect. An effective strategy for preventing diabetic complications has not yet been developed [3,9,10]. Therefore, identifying a potential therapeutic candidate for preventing and delaying the onset of DM and its complications is of great interest.

Emerging studies have focused on naturally occurring phenolic substances to provide a pharmaceutical strategy for treating metabolic disorders [11,12]. Flavonoids are a vast and ubiquitous group of phytonutrients. As a member of the flavonoid group, luteolin (3′, 4′, 5, 7-tetrahydroxyflavone) exhibits a wide spectrum of biological benefits, including anti-inflammatory, anti-oxidative, anti-carcinogenic, anti-allergenic, vasoprotective, and neuroprotective effects, that may be attributed to the presence of diverse chemical entities [13,14,15,16]. In in vitro and in vivo models of ischemic-induced cell injury, luteolin suppresses the secretion of tumor necrosis factor (TNF)-α, interleukin (IL)-6, and inducible nitric oxide synthase (iNOS) in lipopolysaccharide (LPS)-activated macrophages [17,18]. Luteolin also inhibits the neutrophil extracellular traps (NETs) in activated human neutrophils, reduces oxidative stress, and enhances immune defense by inhibiting the production of reactive oxygen species (ROS) [13]. In addition to its radical scavenging capacity, luteolin exerts great potential against multiple malignancies, activating cell-death pathways and cell cycle arrest, blocking angiogenesis and influencing tumor development by mediating insulin-like growth factor (IGF), platelet-derived growth factor (PDGF), and fibroblast growth factor (FGF) [19,20]. Interestingly, luteolin also contributes to anti-diabetic action, owing to its anti-oxidative capacity, as it targets signature cascades of AMP-activated protein kinase (AMPK), growth factors, cytokines, and p53-generated ROS and superoxide dismutase 1 (SOD1) [21,22,23,24].

Thus, luteolin is a promising candidate for ameliorating oxidative stress and inflammation. However, the capacity of luteolin to promote cutaneous wound closure and its interaction with crucial factors involved in delayed wound healing under hyperglycemic conditions has not been clarified. In the present study, we proposed that luteolin restores the cellular redox status and suppresses the inflammatory cascade to exert healing-promoting effects and further explored the detailed mechanistic pathways.

## 2. Materials and Methods

### 2.1. Animals and Experimental Design

Eighteen 8-week-old male Wistar rats weighing 250–300 g each were purchased from BioLASCO Taiwan Co., Ltd. (Taipei, Taiwan). Rats were housed in a controlled environment (23 ± 2 ℃, relative humidity 60%, 12 light/dark cycles) at the Experimental Animal Center of Chung Shan Medical University. The rats were acclimated for 7 days while being fed a laboratory chow diet and receiving water ad libitum. Subsequently, the experimental rats were randomly assigned to three groups: (a) non-DM (NDM) group, (b) DM group, and (c) DM with 100 mg/kg body weight luteolin administered intraperitoneally (DML) group (Santa Cruz, sc-203119C). For induction of diabetes, rats were fasted overnight and injected intraperitoneally with streptozotocin (STZ) (80 mg/kg) (Sigma-Aldrich, St. Louis, MO, USA) freshly dissolved in citrate buffer (pH 4.5). After the rats were fasted for 12 h (from 9 pm to 9 am), the blood collected from the tail vein was recorded to determine the fasting blood glucose on days 1, 5, 7, 10, and 14 of the experiment. After the 14-day treatment period, the animals were fasted overnight, then anesthetized with an overdose of inhaled carbon dioxide. The experimental procedures were approved by the Institutional Animal Care and Use Committee of Chung Shan Medical University on 07-Apr-2020 (IACUC Approval No 2310) and performed according to the ethical guidelines for animal experiments.

### 2.2. Wound Induction

After the fast overnight, the rats were injected intraperitoneally with STZ (80 mg/kg) at 9 am. Two days after the successful induction of hyperglycemia (above 250 mg/dL), wound surgery was performed. The rats were anesthetized with isofluance administered via precision vaporizer. A 15 × 15 mm^2^ incision was made surgically in the skin on the dorsal thorax. For the treatment every day, the uncovered wound was rinsed with normal saline at 9 am before the rats were injected intraperitoneally with Luteolin (100 mg/kg). The wound diameter was determined with a digital caliper over 14 days. In addition, excisional biopsies of the wound were taken for histological and immunohistochemical examination. For mechanistic cascade analysis, tissue samples from the wound area were harvested on day 14 and stored at −80℃ until use. The rate of closure within the wound margins was evaluated on macroscopic images using Image J software.

### 2.3. Histological Analysis

Tissue samples from wound biopsies were fixed in 10% formalin buffered solution and embedded in paraffin, followed by staining with hematoxylin and eosin (H&E). Standard procedures were performed to detect cellular infiltration, re-epithelization and extracellular matrix (ECM) rearrangement [25]. The density of collagen was assessed using Masson’s trichrome stain. Skin tissue samples were photographed under blind conditions using a light microscope (Axioplane 2, Carl Zeiss MicroImaging GmbH, Hamburg, Germany).

### 2.4. Immunohistochemical Staining

Sections were immersed in 0.01 M phosphate-buffered saline (PBS) containing 3% H_2_O_2_/methanol solution for 1 h and washed with 0.01 M PBS (pH 7.4) to eliminate endogenous peroxidase activity. Following rinses in PBS, sections were incubated in blocking medium containing 0.1% Triton X-100, 3% normal goat serum and 2% bovine serum albumin (BSA) for 1 h to block nonspecific binding. The sections were reacted with primary antibody nuclear factor (NF)-κB (p65) (1:200, Santa Cruz Biotechnology, Santa Cruz, CA, USA, sc-8008), TNF-α (1:100, Santa Cruz Biotechnology, sc-52746), IL-1β (1:100, Santa Cruz Biotechnology, sc-52012), IL-6 (1:500, Bioss Antibodies, Woburn, MA, USA, bs-0782R-TR), MMP-9 (1:200, Santa Cruz Biotechnology, sc-13520) and vascular endothelial growth factor (VEGF) (1:200, Santa Cruz Biotechnology, sc-7269) at 4 °C for 48 h. After washing with 0.01 M PBS (pH7.4), sections were placed in 1:200 secondary antibody at room temperature for 2 h. Then, the sections were immersed in 1:100 concentration of avidin-biotin complex (ABC complex, Vector) at room temperature for 1 h with 3,3′ diaminobenzidine (DAB, Sigma, St. Louis, MO, USA) as the chromogen. All reacted sections were rapidly dehydrated through a series of graded alcohols, cleared with xylene and coverslipped with Permount.

### 2.5. Western Blot Analysis

Skin wound tissue samples were homogenized in RIPA buffer and centrifuged at 12,000× *g* at 4 ℃ for 30 min to collect supernatants for Western blot analysis. Total protein concentration was quantified using Bradford assay (Bio-Rad, Hercules, CA, USA) according to the manufacturer’s instructions. Subsequently, Western blotting was performed on SDS-PAGE with electrophoretic transfer to nitrocellulose (NC) papers (Trans-blot; BioRad). NC papers were incubated in 5% BSA at room temperature for blocking. After blocking, there was incubation with specific antibodies MMP-9 (1:500, Santa Cruz Biotechnology, sc-13520), NF-κB (1:500, Santa Cruz Biotechnology, sc-8008), TNF-α (1:200, Santa Cruz Biotechnology, sc-52746), IL-6 (1:500, Bioss Antibodies, bs-0782R-TR), IL-1β (1:200, Santa Cruz Biotechnology, sc-52012), nuclear factor erythroid-2-related factor 2 (Nrf2) (1:1000, Affinity Biosciences, Cincinnati, OH, USA, AF0639), phospho-Nrf2 (1:1000, Affinity Biosciences, Cincinnati, OH, USA, DF7519), SOD1 (1:1000, Thermo Scientific, Rockford, IL, USA, PAI-30195), glutathione peroxidase (GSH-Px) (1:1000, Abcam, Ab22604), and ubiquitin carboxy-terminal hydrolase (UCH)-L1 (1:500, Santa Cruz Biotechnology, sc-271639) at 4 °C overnight. NC papers were washed with tris-buffered saline and 0.1% Tween 20 (TBST) 4 times, then incubated with anti-rabbit HRP-conjugated secondary antibody (1:5000, Sigma). Signals were visualized by chemiluminescence with a Western Blotting Detection Kit (Renaissance kit; NEN, Boston, MA, USA).

### 2.6. Immunofluorescence

Skin wound tissue sections were placed on glass coverslips, then incubated with blocking buffer (0.1% Triton X-100, 3% normal goat serum, and 2% BSA) for 1 h. For determining UCH-L1 expressions, coverslips were incubated with UCH-L1 primary antibody (1:200, Santa Cruz Biotechnology, sc-271639) to localize nerve tissue. Subsequently, the coverslips were washed with PBS and incubated with goat anti-mouse secondary antibody conjugated with Alexa Fluor^®^ 488 (1:200, Thermo Scientific, A-11029) at room temperature for 1 h. Cell nuclei were counterstained with DAPI and mounted on glass slides with Prolong Antifade Reagent (Invitrogen, Carlsbad, CA, USA). Images were examined under fluorescence microscope.

### 2.7. Statistical Analysis

All data are presented as mean ± standard error (SEM). Differences in quantitative results were determined by one-way analysis of variance (ANOVA) followed by post-hoc multiple comparisons using Bonferroni test. Student’s t-test was used to analyze blood glucose levels and related protein expressions. Statistical difference was considered significant when p value was less than 0.05.

## 3. Results

### 3.1. Luteolin Attenuates Blood Glucose Level in STZ-Induced Diabetic Rats

**Figure 1:**
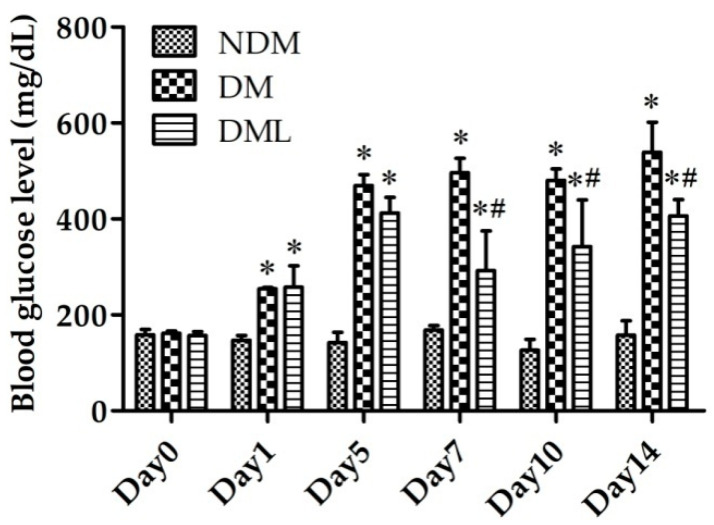
Luteolin ameliorated blood glucose levels in STZ-induced diabetic rats. Changes in blood glucose in STZ-induced diabetic rats supplemented with luteolin during 14-day experimental period. Values of individual groups are presented as mean ± SEM. * *p* < 0.05, compared with NDM group. # *p* < 0.05, compared with DM group. NDM, non-diabetes mellitus; DM, diabetes mellitus; DML, diabetes mellitus treated with luteolin 100 mg/kg body weight, ip.

To explore the in vivo anti-hyperglycemic effects of systemic luteolin administration, STZ-induced diabetic rats were supplemented with luteolin for 14 days. As shown in Figure 1, blood glucose levels were elevated, as incidence of hyperglycemia in DM and DML groups on day 1. We observed persistent hyperglycemia in the DM group, while blood glucose level significantly decreased in the DML group, compared to the DM group, on day 5. The results of this preliminary in vivo evaluation demonstrate the anti-diabetic activity of luteolin in STZ-induced diabetes.

### 3.2. Luteolin Accelerates Wound Re-epithelization and Inflammatory Response in STZ-Induced Diabetic Rats

**Figure 2:**
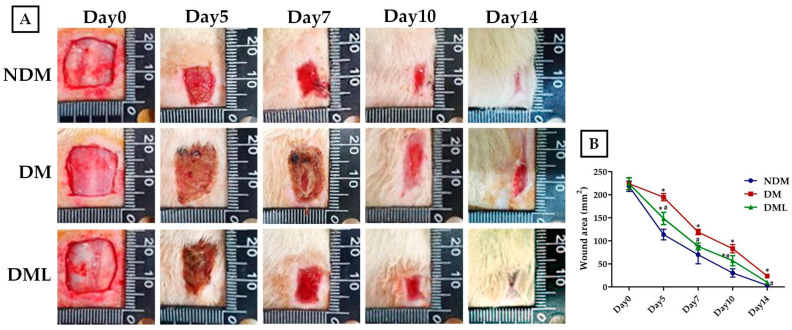
Effects of luteolin on wound healing rate in STZ-induced diabetic rats. Skin wound was generated with 15 mm punch biopsies on the dorsal thorax of STZ-induced diabetic rats and non-diabetic rats. (**A**) Representative images of the skin wound were recorded on days 0, 5, 7, 10, and 14 during the experimental period. (**B**) Quantitative images of the wound margin were obtained by measuring the pixel size. Values of individual groups are presented as mean ± SEM. * *p* < 0.05, compared with NDM group. # *p* < 0.05, compared with DM group.

**Figure 3:**
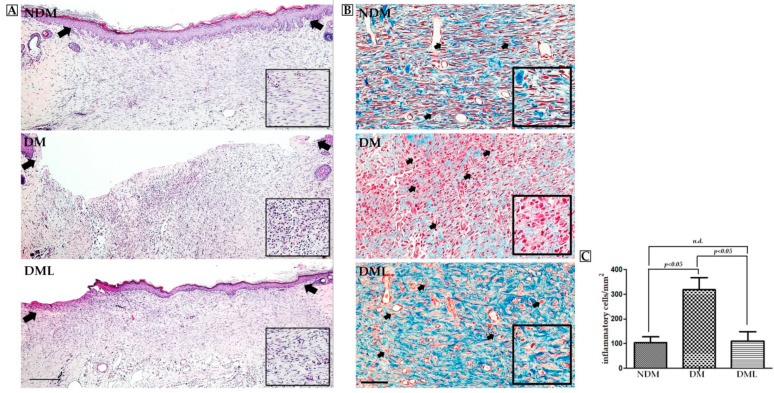
Luteolin accelerates wound re-epithelialization and ameliorated inflammatory cell infiltration. (**A**) Histological sections from the wound site demonstrated complete and incomplete re-epithelialization and collagen deposition on day 14. Delayed re-epithelialization was observed in the DM group (black arrowheads denote re-epithelization of the wound). Scale bars: 300 μm. (**B**) Representative images of skin wounds (Masson trichrome staining) showed loss of infiltrating inflammatory cells in the NDM and DML groups (arrowheads). (**C**) Average numbers of inflammatory cells were determined using Image J software. Values of individual groups are presented as mean ± SEM. Difference was considered significant when *p* < 0.05. Scale bars: 200 μm.

To explore whether systemic luteolin administration improves wound healing in diabetic rats, a 15 × 15 mm^2^ wound was created and wound diameter was evaluated periodically during the experimental period. On day 14, the wound in the NDM group had completely healed. In the DM group, there remained a small incompletely healed wound. The wound surface significantly decreased following luteolin supplementation (DML group) in comparison with the DM group (Figure 2A). Moreover, continuous wound contraction was delayed in the DM group. Systemic luteolin administration improved delayed wound healing over 14 days (Figure 2B). On day 14, wound tissue was sampled and assessed for histological change. Microscopic evaluation with H&E staining showed that on day 14, there were large numbers of fibroblasts in the wounds of the NDM and DML groups with good re-epithelialization. However, the fibers in the DM group were in a disordered arrangement with poor re-epithelialization (Figure 3A). Masson staining showed deposition of collagen fibers at the wound site. The wounds in the DM group showed severely disordered collagen fiber arrangement and massive infiltration of inflammatory cells (black arrow). Wounds in the NDM and DML groups showed better and denser collagen fiber arrangement (Figure 3B). Compared with the DM group, NDM and DML groups showed fewer inflammatory cells (Figure 3C).

### 3.3. Luteolin Attenuates NF-κB-Induced Inflammatory Response and Nrf2-Mediated Oxidative Response in STZ-Induced Diabetic Rats

**Figure 4:**
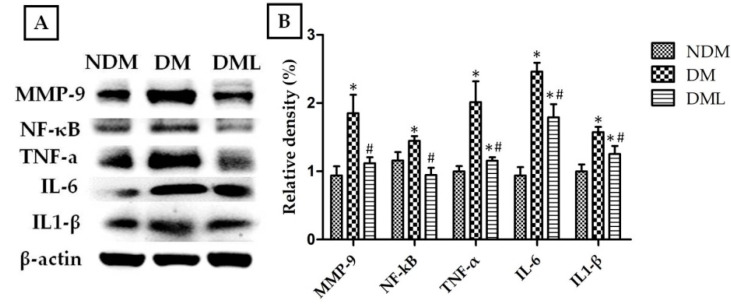
Effects of luteolin on protein expression levels of inflammatory factors of skin wound on day 14. (**A**) Tissues were homogenized in RIPA buffer and subjected to immunoblotting with anti-MMP-9, anti-NF-κB, anti-TNF-α, anti-IL-6, and anti-IL1-β antibodies. (**B**) Quantitative results of MMP-9, NF-κB, TNF-α, IL-6, and IL1-β protein levels were adjusted to β-actin protein level. Values of individual groups are presented as mean ± SEM. * *p* < 0.05, compared with the NDM group. # *p* < 0.05, compared with the DM group.

**Figure 5:**
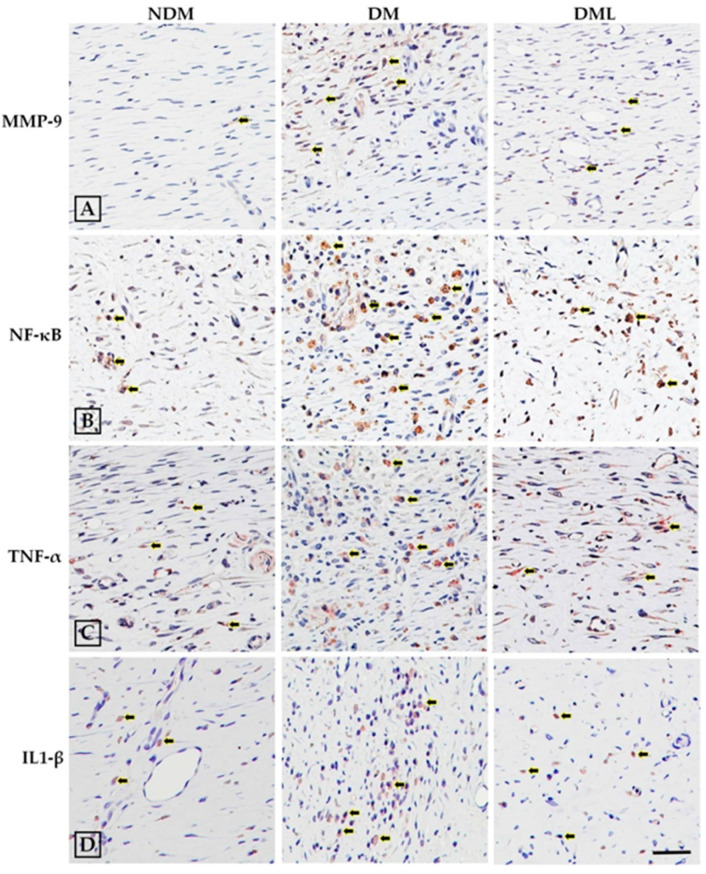
Luteolin ameliorated inflammatory factors. To determine the inflammatory factors, tissue sections obtained from skin wound on day 14 were analyzed using immunohistochemistry for (**A**) MMP-9, (**B**) NF-κB, (**C**) TNF-α, and (**D**) IL1-β. Brown areas indicate positive staining (arrowheads). Scale bars: 100 μm.

**Figure 6:**
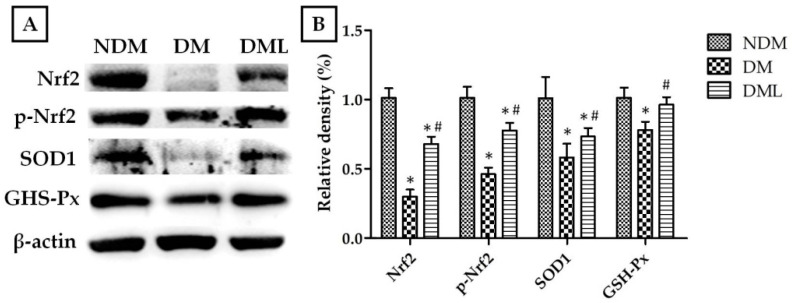
Effects of luteolin on protein expression levels of oxidative enzymes of the skin wound on day 14. (**A**) Tissues were homogenized in RIPA buffer, subjected to immunoblotting with anti-Nrf2, anti-phospho-Nrf2, anti-SOD1, and anti-GSH-Px antibodies. (**B**) Quantitative results of Nrf2, phospho-Nrf2, SOD1, and GSH-Px protein levels were adjusted to β-actin protein level. Values of individual groups are presented as mean ± SEM. * *p* < 0.05, compared with the NDM group. # *p* < 0.05, compared with the DM group.

The cells infiltration of the observed wound is a typical sign of inflammation. Therefore, we explored whether accelerated wound healing in diabetic rats systemically administered with luteolin may be due to modulation of the inflammatory response. As shown in Figure 4, the histological analysis of STZ-induced diabetic rats systemically administrated with luteolin demonstrates a significant decrease in inflammatory cell infiltration when compared with the DM group. Compared with NDM group, immunoblotting results revealed high levels of inflammatory markers, including TNF-α, IL-6, and IL1-β, as well as MMPs (MMP-9), with activation of transcription factor NF-κB in the DM group which was attenuated by luteolin supplementation (Figure 4). Similarly, histological evaluation (Figure 3A) of skin wounds over 14 days confirmed full healing with low levels of TNF-α, IL1-β, and MMP-9 through downregulation of NF-κB in the NDM and DML groups, in contrast to the DM group (Figure 5). As impaired wound healing also induces oxidative stress, we further explored whether luteolin supplementation in STZ-induced diabetic rats modulates oxidative stress response. As shown in Figure 6, immunoblotting analysis showed that luteolin reversed the effects of reduced SOD1 and GSH-Px expressions, as well as Nrf2 phosphorylation in the DML group. 

### 3.4. Luteolin Accelerates Angiogenesis and Neuronal Regeneration in STZ-Induced Diabetic Rats

**Figure 7:**
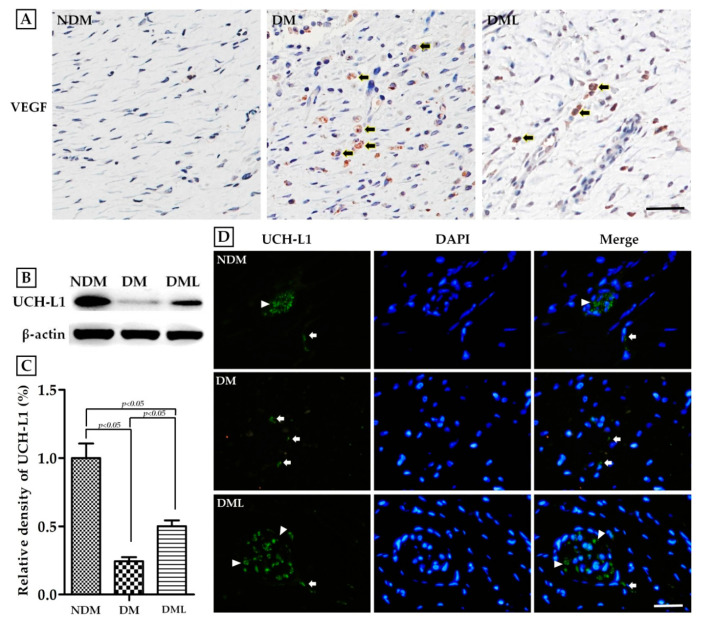
To determine the completeness of healing, tissue sections were obtained from the skin wound on day 14. (**A**) immunohistochemistry for VEGF. Brown areas indicate positive staining (arrowheads). (**B**) Tissues were homogenized in RIPA buffer and subjected to immunoblotting with anti-UCH-L1. (**C**) Quantitative results of UCH-L1 protein levels were adjusted to β-actin protein level. (**D**) Tissue sections were stained for UCH-L1 by immunofluorescence. White triangle denote the locations of UCH-L1-expressing nerve bundles. White arrowheads denote the locations of UCH-L1-expressing nerve fibers. Scale bars: 100 μm.

To corroborate the acceleration of the healing process in diabetic rats systemically administered with luteolin, we examined angiogenesis capacity and nerve regeneration in skin wound biopsy samples. The protein expression of VEGF was considerably higher in the DM group than in the NDM group, whereas systemic luteolin administration resulted in marked decreases in VEGF expression (Figure 7A). Immunofluorescence analysis with an antibody to the neuron cell specific marker UCH-L1 revealed higher UCH-L1-positive structure presenting within the section of skin wound biopsy in the DML group than in the DM group (Figure 7D). Similar effects were observed on immunoblotting analysis (Figure 7B,C).

## 4. Discussion

The global prevalence of DM has risen dramatically over the past few decades, resulting in high socio-economic burden. Chronic non-healing wounds are troublesome and are most common complications of diabetes that contribute to lower extremity amputations and increased morbidity and mortality rates. Multifactorial pathogenesis is related to delayed wound healing, such as impaired glucose metabolism and both macrovascular and microvascular diseases. An STZ-induced diabetic animal model has commonly been used to explore diabetes-related complications including retinopathy, nephropathy, and foot ulcers owing to similar pathogenesis to human type 1 diabetes. Rodent models have been well documented in the study of healing of excisional wounds in diabetic subjects, with straightforward evidence and process of re-epithelialization, angiogenesis and nerve regeneration.

Luteolin is a promising natural bio-substance with pharmacological properties that provides significant protection against inflammation and oxidative stress. These effects may be attributed to several mechanisms. Structure–activity relationship studies have predicted the structural specificity of hydroxyl moieties at A and B rings of the luteolin structure and a C2-C3 double bond in conjugation with an oxo group at C4 position that might exert free radical-scavenging, anti-inflammatory, and anti-oxidant activities [26,27]. Accumulated research has shown that long-term treatment with luteolin improves pancreas β-cell dysfunction and regulates glucose-stimulated insulin secretion in both in vivo and in vitro diabetic models [28,29]. In the present study, we explored the hypoglycemic effect of luteolin on STZ-induced diabetic rats. In the DML group, the results showed that systemic luteolin administration significantly reduced blood glucose levels from day 7 to 14, compared to the DM group. Consistent with the findings of previous studies, luteolin rescued pancreatic dysfunction and promoted β-cell regeneration for glucose metabolism in the rats’ pancreatic carcinoma cell line [30]. As luteolin is evolving as a potential anti-inflammatory and anti-oxidative stress bioflavonoid, understanding the complete mechanistic action of luteolin in the promotion of wound healing is a prerequisite for research on diabetes therapy.

Wound closure is considered the sum of epithelization and proceeds from wound edge toward the migration of fibroblasts at the original wound area. Impaired skin wound healing is a key manifestation of diabetes. To illustrate wound contraction, an appropriate skin wound model should be carefully performed. A preliminary study of flavonoid extracts from M.annua Linn. leaves containing luteolin demonstrated that 0.5% w/w has potential benefits in improving impaired wound healing in STZ-induced diabetic rats on the basis of traditional folk remedies [31]. This is similar to our results that systemic luteolin administration improved delayed wound healing on day 5 following wound induction with a significant reduction in skin wound area in the DML group compared to the DM group. Initially, the wound healing process starts from damaged blood vessels and microbial pathogen invasion at the wound site, rapidly followed by acute inflammatory response mediated by pro-inflammatory cells including neutrophils and macrophages and the production of cytokines. Infiltration of inflammatory cells were typically found to increase in the DM group, while the luteolin-treated rats revealed a significant decrease in the DML group. Importantly, phenolic substances possess wide-ranging pharmacological properties including anti-oxidative stress, anti-fibrosis, anti-tumor, anti-microbial infection, and anti-inflammatory activities and, thus, exerted valuable medicinal applications. For example, quercetin enhances fibroblast migration and proliferation, while inhibiting fibrotic scarring, which contributes to accelerated wound closure [32]. Interestingly, comparative research on the composition of phenolic substances in medicinal plants with abundant luteolin has shown a significant association between total anti-oxidant activity and wound healing in an in vitro NIH-3T3 fibroblast model [31]. This is similar to our results, that systemic luteolin administration promotes wound re-epithelialization with well-organized fibroblast arrangement. Furthermore, chronic inflammation is due to prolonged inflammatory cell migration in the wound area, thus limiting fibroblast arrangement and collagen synthesis, which subsequently leads to impairment of the healing mechanism. In vivo studies on rodents with diabetes have indicated differing expression levels of VEGF which are associated with impaired vascularization, failed wound contraction and reduced epidermal re-epithelization and formation [33,34]. In the present study, immunohistochemistry and H&E staining revealed that VEGF expression and local inflammatory infiltration are reduced in the DML group when compared with the DM group. Based on these aforementioned data, luteolin shows anti-inflammatory and anti-oxidative effects in the diabetic wound rat model.

Persistent oxidative stress stimulates the activation of NF-κB-mediated inflammatory cytokines production, including TNF-α and ILs, which leads to chronic inflammation with requirement for NF-κB during diabetic wound healing [35]. Consequently, in the present study, immunoblotting and immunohistochemistry results revealed that NF-κB, TNF-α, IL6, and IL1-β protein expressions dramatically increase after wound creation, with significant reversal after 14-day luteolin treatment in the DML group, consistent with the findings that luteolin improves fibroblast arrangement and inflammatory cell infiltration. In addition to reducing levels of NF-κB, TNF-α, IL-6, and IL1-β proteins, luteolin lowers MMP-9 expression, which is also implicated in the regulation of angiogenesis and tissue remodeling during diabetic wound healing. Interestingly, this result was consistent with our findings, that increased proangiogenic factor VEGF stimulates new vessels to sprout at the wound site as evidence of delayed wound contraction, and was reversed by luteolin treatment. Moreover, it has been previously confirmed that high expression levels of VEGF in early stage of wound healing promote vascular permeability, while low levels of VEGF were found in epithelialized wound [36]. Accumulated evidence from in vivo, in vitro, and in silico studies reveals that phenolic compounds improve inflammation and oxidative stress via modulation of various genes and relevant signal transduction involved in wound healing. Latha demonstrated the antidiabetic effects of beleric myrobalan phenolic extract containing gallic acid, including regeneration of pancreatic β-cell function and stimulation of insulin secretion in STZ-induced diabetic rat model [37]. We speculated that antidiabetic action of luteolin is due to its antioxidative stress potential, which provides an appropriate environment for diabetic wound healing. Moreover, sustained oxidative stress might facilitate NF-κB activation and prolong inflammation. Therefore, in this regard, further investigations are necessary to clarify the molecular mechanisms by which oxidative stress is regulated by luteolin. As expected, highly significant increases in the expression levels of SOD1 and GHS-Px were observed in the DML group compared to the DM group. Luteolin has been previously reported to exert cardiovascular and neuronal protection via inhibition of NF-κB-mediated inflammatory response and to trigger Nrf-2 activation, which enhances transcriptional activity of antioxidant genes in rodent models [38,39]. Indeed, the present data shows that luteolin enhances SOD1 and GHS-Px expressions by promoting phosphorylation of Nrf-2.

The nervous system is responsible for the innervation of skin vasculature and the detection of tissue injury. Diabetic wound healing involves sensitization and regeneration of nociceptive neurons, modulated by UCH-L1, which are involved in ECM synthesis and re-epithelization [40,41,42]. It has been reported that UCH-L1 is exclusively found in neurons and plays a role in neuronal regeneration within tissue repair [43,44,45]. Therefore, UCH-L1 may be a biomarker of tissue damage and may indicate the recovery status in diabetic wound healing. Results of immunofluorescence staining revealed a significant decrease in UCH-L1 fluorescence intensity in the DM group when compared with the NDM group, with an obvious increase after luteolin treatment in the DML group.

In this study, nevertheless, there are some advantages as well as some limitations. Although it is not new for the wound healing applied with luteolin, the main study is for the systemic effect instead of the local effects. Therefore, this study uses an intraperitoneal injection of luteolin to reduce the oxidative pressure and inflammation in hyperglycemic rats to improve the development of wound healing.

## 5. Conclusions

**Figure 8:**
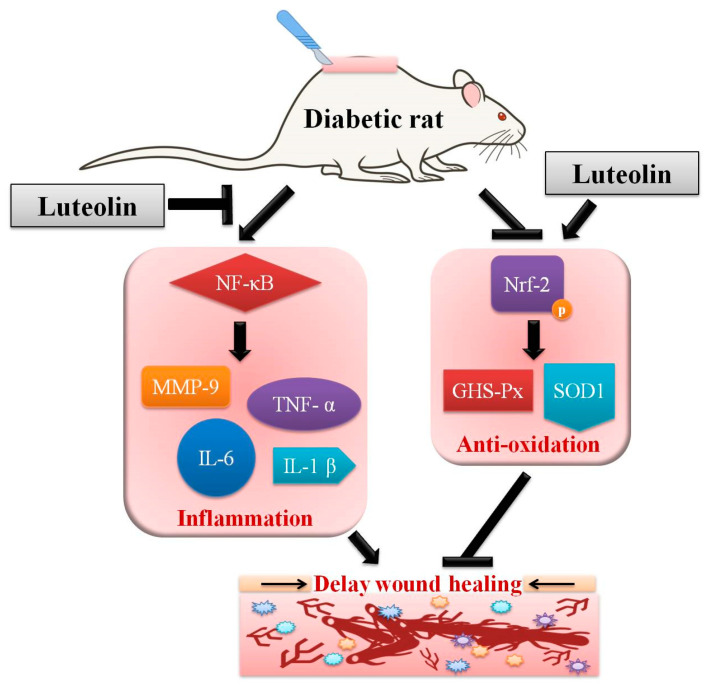
Schematic representation of action by luteolin on impaired wound healing in STZ-induced rats. Luteolin improves diabetic wound healing by inhibiting inflammation and improving antioxidative capacity, which associates with NF-κB-mediates MMP-9, IL-6, TNF-α, and IL-1β downregulation, and Nrf-2-mediates GHS-Px and SOD1 upregulation. Arrows: improve or increase. T-bar: inhibit.

The present investigation was the first to characterize the effect of systemic luteolin administration in diabetes-impaired cutaneous healing in STZ-induced diabetic rats, and further explored the associated regulatory mechanism. The improvement in the delayed wound healing process may be related to the anti-hyperglycemic, anti-inflammatory, and anti-oxidative effects of luteolin in rats with diabetes. Our findings also provide a new insight into the mechanisms of luteolin that are involved in improving wound repair. Luteolin appears to downregulate inflammatory mediators by inhibiting NF-κB expression, while upregulating antioxidant enzymes through activation of Nrf-2 phosphorylation (Figure 8). Moreover, we also provided biomarker of angiogenesis and neuronal regeneration for indicate recovery status in diabetic wound healing. Thus, luteolin is a promising candidate for treating diabetic wound injury.

## Abbreviations

STZ	streptozotocin
MMP-9	matrix metalloproteinase-9
TNF-α	tumor necrosis factor-α
IL-6	interleukin-6
SOD1	superoxide dismutase
GSH-Px	glutathione peroxidase
Nrf-2	nuclear factor erythroid 2-related factor
VEGF	vascular endothelial growth factor
UCH-L1	ubiquitin carboxy-terminal hydrolase-1
